# Supplementary material for: Dowry demand, perception of wife-beating, decision making power and associated partner violence among married adolescent girls: A cross-sectional analytical study in India
Source: PLoS One. 2024 Oct 24;19(10):e0312341. doi: 10.1371/journal.pone.0312341 (PMC11501023; doi:10.1371/journal.pone.0312341)
Supplement: S1 File — S1 Table. Unadjusted estimates (Crude ORs) from logistic regression analysis of types of violence among married adolescent girls aged 15–19 years. (DOCX) [file pone.0312341.s001.docx]

**Table-S1.** Unadjusted estimates (Crude ORs) from logistic regression analysis of types of violence among married adolescent girls aged 15-19 years

| **Background characteristics** | **Emotional Violence** | **Physical Violence** | **Sexual Violence** | **Any Violence** |
| --- | --- | --- | --- | --- |
|  | **OR (CI 95%)** | **OR (CI 95%)** | **OR (CI 95%)** | **OR (CI 95%)** |
| **Dowry demanded by in-laws** |  |  |  |  |
| No |  |  |  |  |
| Yes | 4.78 [3.8-6.02]*** | 3.75 [3.01-4.67]*** | 2.41 [1.93-3.01]*** | 3.95 [3.27-4.75]*** |
| **Perception over wife-beating** |  |  |  |  |
| Not justified |  |  |  |  |
| Justified | 1.44 [1.15-1.8]*** | 1.61 [1.3-2]*** | 1.96 [1.59-2.42]*** | 1.67 [1.37-2.04]*** |
| **Decision-making on going to work** |  |  |  |  |
| Herself or jointly with others |  |  |  |  |
| Others Only | 0.65 [0.52-0.81]*** | 1.05 [0.88-1.24] | 0.84 [0.69-1.03]* | 0.7 [0.58-0.84]*** |
| **Decision-making on household purchases** |  |  |  |  |
| Herself or jointly with others |  |  |  |  |
| Others Only | 0.6 [0.48-0.76]*** | 0.88 [0.74-1.04] | 0.84 [0.67-1.04] | 0.64 [0.53-0.78]*** |
| **Paid work (last 12 months)** |  |  |  |  |
| Yes |  |  |  |  |
| No | 0.89 [0.68-1.17] | 0.64 [0.5-0.82]*** | 0.69 [0.55-0.86]*** | 0.72 [0.58-0.89]*** |
| **Marital duration (in years)** |  |  |  |  |
| ≤1 |  |  |  |  |
| 2-3 | 2 [1.61-2.49]*** | 2.09 [1.7-2.56]*** | 1.02 [0.85-1.23] | 1.51 [1.29-1.77]*** |
| ≥4 | 4.15 [3.04-5.67]*** | 3.25 [2.47-4.28]*** | 1.17 [0.91-1.51] | 2.66 [2.05-3.45]*** |
| **Age groups (in years)** |  |  |  |  |
| 15-17 |  |  |  |  |
| 18-19 | 1 [0.82-1.23] | 1.3 [1.07-1.58]** | 0.83 [0.69-0.99]** | 1 [0.83-1.2] |
| **Education level (in years)** |  |  |  |  |
| No education |  |  |  |  |
| 1-7 years | 0.99 [0.72-1.35] | 0.82 [0.66-1.03]* | 1.01 [0.79-1.29] | 1.09 [0.86-1.37] |
| 8-9 years | 0.79 [0.63-0.99]** | 0.64 [0.5-0.81]*** | 0.88 [0.7-1.1] | 0.84 [0.69-1.01]* |
| 10 & above | 0.44 [0.34-0.58]*** | 0.32 [0.25-0.42]*** | 0.58 [0.45-0.76]*** | 0.51 [0.41-0.64]*** |
| **Age of Spouse** |  |  |  |  |
| ≤ 21 years |  |  |  |  |
| 22-24 years | 1.11 [0.84-1.46] | 0.98 [0.78-1.23] | 0.74 [0.61-0.91]** | 0.99 [0.8-1.22] |
| 25+ years | 1.1 [0.88-1.37] | 1.21 [0.99-1.5]* | 1.01 [0.78-1.31] | 1.13 [0.92-1.38] |
| Don't Know | 1.47 [1.08-1.99]** | 1.42 [0.98-2.06]* | 1.26 [0.9-1.75] | 1.52 [1.13-2.04]** |
| **Place of residence** |  |  |  |  |
| Urban |  |  |  |  |
| Rural | 1.31 [1.03-1.67]** | 1.04 [0.84-1.31] | 1.18 [0.91-1.54] | 1.25 [1-1.56]* |
| **Caste** |  |  |  |  |
| SC/ST |  |  |  |  |
| OBC | 0.92 [0.75-1.12] | 0.72 [0.59-0.88]*** | 0.66 [0.52-0.83]*** | 0.85 [0.7-1.02]* |
| Others | 0.41 [0.29-0.57]*** | 0.42 [0.3-0.59]*** | 0.57 [0.41-0.8]*** | 0.47 [0.36-0.62]*** |
| **Religion** |  |  |  |  |
| Hindu |  |  |  |  |
| Non-Hindu | 0.89 [0.68-1.17] | 0.89 [0.69-1.13] | 0.96 [0.72-1.27] | 0.99 [0.8-1.23] |
| **Wealth quintile** |  |  |  |  |
| Poorest |  |  |  |  |
| Poorer | 0.99 [0.77-1.27] | 0.86 [0.67-1.1] | 0.91 [0.7-1.18] | 0.89 [0.7-1.13] |
| Middle | 0.93 [0.7-1.23] | 0.84 [0.64-1.09] | 0.86 [0.65-1.14] | 0.9 [0.68-1.18] |
| Richer | 0.82 [0.57-1.18] | 0.59 [0.45-0.79]*** | 0.73 [0.54-0.98]** | 0.81 [0.6-1.09] |
| Richest | 0.58 [0.42-0.81]*** | 0.48 [0.34-0.67]*** | 0.64 [0.46-0.88]** | 0.56 [0.42-0.74]*** |
| **State** |  |  |  |  |
| Uttar Pradesh |  |  |  |  |
| Bihar | 2.2 [1.74-2.8]*** | 1.28 [1.03-1.59]** | 1.34 [1.02-1.75]** | 1.57 [1.27-1.95]*** |

***p<0.001; **p<0.05; *p<0.10; aOR: Adjusted Odds Ratio; CI: Confidence Interval
