# Supplementary material for: Dowry demand, perception of wife-beating, decision making power and associated partner violence among married adolescent girls: A cross-sectional analytical study in India
Source: PLoS One. 2024 Oct 24;19(10):e0312341. doi: 10.1371/journal.pone.0312341 (PMC11501023; doi:10.1371/journal.pone.0312341)
Supplement: S2 File — S2 Table. Adjusted estimates (adjusted ORs) from logistic regression analysis of types of violence among married adolescent girls aged 15–19 years. (DOCX) [file pone.0312341.s002.docx]

**Table-S2.** Adjusted estimates (adjusted ORs) from logistic regression analysis of types of violence among married adolescent girls aged 15-19 years

| **Background characteristics** | **Emotional Violence** | **Physical Violence** | **Sexual Violence** | **Any Violence** |
| --- | --- | --- | --- | --- |
|  | **aOR (CI 95%)** | **aOR (CI 95%)** | **aOR (CI 95%)** | **aOR (CI 95%)** |
| **Dowry demanded by in-laws** |  |  |  |  |
| No |  |  |  |  |
| Yes | 4.32[3.42-5.45]*** | 3.62[2.88-4.54]*** | 2.32[1.9-2.85]*** | 3.64[3.05-4.35]*** |
| **Perception over wife-beating** |  |  |  |  |
| Not justified |  |  |  |  |
| Justified | 1.3[1.04-1.62]** | 1.36[1.07-1.73]** | 1.82[1.48-2.24]*** | 1.55[1.27-1.88]*** |
| **Decision-making on going to work** |  |  |  |  |
| Herself or jointly with others |  |  |  |  |
| Others Only | 0.69[0.54-0.88]*** | 1.06[0.84-1.33] | 0.84[0.67-1.05] | 0.74[0.59-0.92]** |
| **Decision-making on household purchases** |  |  |  |  |
| Herself or jointly with others |  |  |  |  |
| Others Only | 0.69[0.54-0.89]*** | 0.91[0.72-1.16] | 0.88[0.69-1.12] | 0.72[0.58-0.9]*** |
| **Paid work (last 12 months)** |  |  |  |  |
| Yes |  |  |  |  |
| No | 1.1[0.81-1.49] | 0.78[0.58-1.04]* | 0.8[0.63-1.03]* | 0.83[0.65-1.06] |
| **Marital duration (in years)** |  |  |  |  |
| ≤1 |  |  |  |  |
| 2-3 | 1.87[1.48-2.37]*** | 2.01[1.62-2.48]*** | 0.94[0.77-1.14] | 1.41[1.19-1.68]*** |
| ≥4 | 3.34[2.51-4.44]*** | 2.46[1.85-3.27]*** | 0.92[0.7-1.21] | 2.09[1.63-2.68]*** |
| **Age groups (in years)** |  |  |  |  |
| 15-17 |  |  |  |  |
| 18-19 | 0.87[0.68-1.09] | 1.23[0.98-1.55]* | 0.91[0.74-1.12] | 0.93[0.76-1.16]* |
| **Education level (in years)** |  |  |  |  |
| No education |  |  |  |  |
| 1-7 years | 1.07[0.84-1.38] | 0.9[0.69-1.18] | 1.13[0.88-1.46] | 1.23[1-1.51]* |
| 8-9 years | 1.07[0.83-1.39] | 0.84[0.66-1.07] | 1.09[0.86-1.38] | 1.12[0.92-1.38] |
| 10 & above | 0.64[0.49-0.85]*** | 0.46[0.34-0.61]*** | 0.79[0.6-1.04] | 0.73[0.58-0.92]** |
| **Age of Spouse** |  |  |  |  |
| ≤ 21 years |  |  |  |  |
| 22-24 years | 0.96[0.77-1.2] | 0.89[0.7-1.14] | 0.77[0.62-0.96]** | 0.9[0.75-1.09] |
| 25+ years | 0.9[0.69-1.16] | 1.03[0.82-1.3] | 1.04[0.79-1.38] | 1.01[0.8-1.28] |
| Don't Know | 1.17[0.83-1.65] | 1.13[0.77-1.67] | 1.13[0.8-1.59] | 1.34[0.97-1.85] |
| **Place of residence** |  |  |  |  |
| Urban |  |  |  |  |
| Rural | 0.93[0.74-1.17] | 0.8[0.63-1.02]* | 1[0.77-1.3] | 1.01[0.81-1.26] |
| **Caste** |  |  |  |  |
| SC/ST |  |  |  |  |
| OBC | 0.92[0.74-1.13] | 0.78[0.62-0.98]** | 0.67[0.52-0.86]*** | 0.87 [0.72-1.06] |
| Others | 0.53[0.37-0.76]*** | 0.57[0.39-0.84]** | 0.68[0.48-0.99]** | 0.59 [0.43-0.80]*** |
| **Religion** |  |  |  |  |
| Hindu |  |  |  |  |
| Non-Hindu | 1.17[0.87-1.58] | 0.97[0.74-1.27] | 1.14[0.84-1.55] | 1.22 [0.97-1.53] |
| **Wealth quintile** |  |  |  |  |
| Poorest |  |  |  |  |
| Poorer | 1.31[0.99-1.74] | 1.06[0.8-1.4] | 1.06[0.79-1.43] | 1.11 [0.84-1.47] |
| Middle | 1.24[0.91-1.68] | 1.1[0.82-1.46] | 1.03[0.75-1.41] | 1.16 [0.87-1.56] |
| Richer | 1.05[0.78-1.42] | 0.83[0.58-1.18] | 0.95[0.68-1.32] | 1.05 [0.79-1.4] |
| Richest | 0.99[0.68-1.44] | 0.87[0.6-1.26] | 0.96[0.66-1.38] | 0.93 [0.67-1.29] |
| **State** |  |  |  |  |
| Uttar Pradesh |  |  |  |  |
| Bihar | 1.5[1.17-1.93]*** | 0.85[0.68-1.06] | 1.18[0.91-1.53] | 0.85 [0.68-1.06] |

***p<0.001; **p<0.05; *p<0.10; aOR: Adjusted Odds Ratio; CI: Confidence Interval; The aOR are adjusted for Paid work (last 12 months), Marital duration (in years), Age groups (in years), Education level (in years), Place of residence, Caste, Religion, Wealth quintile, State
