# Supplementary material for: Dowry demand, perception of wife-beating, decision making power and associated partner violence among married adolescent girls: A cross-sectional analytical study in India
Source: PLoS One. 2024 Oct 24;19(10):e0312341. doi: 10.1371/journal.pone.0312341 (PMC11501023; doi:10.1371/journal.pone.0312341)
Supplement: Attachment — Submitted filename: Response to reviewers_rev1.docx [file pone.0312341.s003.docx]

**Response to comments**

**Reviewer #1:**

The research and resulting manuscript is very well done. Please complete minor editorial changes (e.g. data should always be plural, "data are" not "is").

**Response: Thanks for the suggestion. Changes have been made.**

Also, is there a possibility for comparison of data from other Indian states? That would be an interesting next step.

**Response: The survey was done in only these two states: Bihar and Uttar Pradesh therefore author unable to compare data with other Indian states.**

**Reviewer #2:**

Referee Report on “Dowry demand and associated partner violence among married adolescent girls: A cross-sectional analytical study in India”

This paper explores the relationship between dowry demand and domestic violence in two states in India. The methodology is descriptive, and the authors find that the prevalence of domestic violence was higher amongst girls who reported that dowry was demanded by their husbands. While, an important topic with nuanced relationships this paper does not make a sufficient enough contribution beyond the existing literature. Here are the main reasons why:

1. The paper is based on data from the UDAYA project. The sole advantage of this data over more comprehensive and nationally representative surveys such as the DHS which records domestic violence, is that an additional question regarding dowry demand was asked. Why did the authors not use the India Human Development Survey (IHDS) panel dataset which has even more detailed dowry and domestic violence information and more coverage with regards to age and geography?

**Response: Dear reviewer, I agree with your comment that India Human Development Survey (IHDS) panel dataset which has even more detailed dowry and domestic violence information and more coverage with regards to age and geography. However, there are certain limitations i.e., the data is very old (2004-05 and 2011-12), therefore the findings cannot be generalised in today’s context. The data was also not available at district level. The authors used UDAYA data because firstly, the authors aim to determine the association between IPV and dowry among adolescent girls. Secondly; in spite of the fact that the data was available only for two states, the sample is representative and also the estimates are of recent times which can be generalized for UP and Bihar.**

**Still authors on the suggestion of reviewer checked IHDS data, it was found that the sample was too low for 15-19 age group females and additionally, the prevalence of dowry was 100%. Therefore, analysing the relationship was not possible.**

2. The authors are particularly interested in examining this amongst adolescent girls, but the paper lacks any motivation as to why this would be interesting and relevant to examine.

**Response: The ‘adolescent girls’ is a vulnerable group with lack of awareness, maturity and need more intervention. Adolescence therefore, is also a period of increased vulnerability to several types of violence. These are mentioned in the revised version.**

3. In the subsection labelled “Data,” the authors elaborate on how they select their analytical sample. However, the numbers do not add up. According to the information provided, the analytical sample of married girls from both states should be 5206 (1798 + 3408) and not 5226. Similarly, the number for the effective sample size do not add up either and the number of observations used for the estimations are not provided in the tables.

**Response: In page number 8 there was an error it was 313 cases were removed.**

**We have to provide the N in other tables.**

**Bihar sample**

Married women was 3408 samples and if we remove 226 then 3182 which was matching our tables similarity UP states also.

226 + 87🡪313. The cases for which Gauna was not formed were removed from the sample (313 cases)

**UP sample**

| **State** |  |  |
| --- | --- | --- |
| Uttar Pradesh | 35.4 | 1,711 |
| Bihar | 64.6 | 3,182 |
| Total | 100.0 | 4893 |

4. The equation in the subsection titled “Statistical Analysis” is not explained at all and there is no discussion of the perils of using a logistic regression to estimate this relationship or that of sample selection (the worst cases of domestic violence are likely not captured by the sample) which are surely important considerations when examining this relationship. Furthermore, there is no discussion of the endogeneity of dowry demanded which is certain to make this estimate biased.

**Response: Dear reviewer, the data do not capture the worst cases of domestic violence. This is now mentioned in the limitation. The equation is explained in the revised version. The issue of endogeneity was looked upon before the analysis; however, it was not mentioned in the manuscript. The authors now have mentioned the same in the manuscript.**

5. Despite these shortcomings, there are some very crucial claims made by the paper which are unsubstantiated. For instance, the sentence “In the process of empowering women, the study indicates that parents appear to be replacing property inheritance to their daughters by giving them alternative transfers in the form of higher dowries.” The authors do not have any information on inheritance and do not present any results for such a replacement. ‘

**Response: The sentence is modified. The statement is related to a previous study which partly explains the less violence among empowered adolescent girls in our study which was mentioned in the next line.**

6. Another such claim in the Conclusion section is “The results presented in this study suggest that policies that ensure equal inheritance and property rights for women and programs that help women retain equal power and say in their families may be necessary to reduce their vulnerability to domestic violence.” The authors have presented no evidence to support this claim.

**Response: Thank you for pointing this. The statements have been revised accordingly.**
