# Supplementary material for: Dowry demand, perception of wife-beating, decision making power and associated partner violence among married adolescent girls: A cross-sectional analytical study in India
Source: PLoS One. 2024 Oct 24;19(10):e0312341. doi: 10.1371/journal.pone.0312341 (PMC11501023; doi:10.1371/journal.pone.0312341)
Supplement: Attachment — Submitted filename: Response to reviewers__Rev2.docx [file pone.0312341.s004.docx]

**Response to comments**

**Reviewer #3:**

This paper examines the relationship between dowry demands and IPV among adolescent girls. I have a few suggestions for improvement:

1) Throughout the paper, I think the language of risk factors need to be changed to correlates since this is a cross sectional study. Also calling a paid job and marital duration as risk factors could present them as factors that need to be corrected for when findings on employment among women related to IPV in India are mixed. The empowerment of women can also sometimes protect women. Being in marriage for a long time can also be a reason why women are facing violence. Authors elaborate on this in the discussion but I think correcting the language early on would be better. This could be changed in the abstract and other areas of the paper.

**Response: The amendments are made as per suggestion.**

2) In introduction, you could include how you define intimate partner violence- method section discusses that but will be good for the reader to know early on.

**Response: The definition of IPV is provided in the introduction section of the revised version.**

3) Methods: the sentence on page 19 needs to be corrected--"For instance, married girls faced more violence to increase the length of the marriage."

**Response: The statement is modified for better understanding.**

4) Discussion- this section could be strengthened with discussing how early marriage in adolescence can enhance risk and the practice and policy implications of early marriage

**Response: Dear reviewer, thank you for the comment. Since the current study focused on married girls aged 15 to 19 years and not above that, the authors feel that discussing on early marriage will be superficial. Even, the age groups of 15-17 and 18-19 years showed no statistically significant association with most of the outcome variables. However, this is acknowledged in detail in limitations of the study with the suggestion of future studies on this aspect.**

**Reviewer #4:**

This is an interesting paper which explores the relationship between demands for dowry and domestic violence focusing on young women which is an important topic. However, the paper raises a number of questions and comments – which if addressed might make for an interesting contribution to the literature in this field.

1. The hypothesis that dowry demands are associated with increased violence against women is neither new nor particularly helpful, unless the authors explain what their study contributes to existing knowledge and why it is important.

**Response: The last paragraph of the introduction section is revised accordingly and added the potential contribution of the study.**

2. The conceptual framework is a bit simplistic – it simply connects a number of facts and says they lead to increased violence. It is not clear if all the elements in the first box lead to all the elements in the second box, and regardless they lead to violence? I would have thought that some of the factors in the second box would be protective factors, i.e. paid work and decision making autonomy – or are the authors proposing that despite the existence of these facts – that all of them are necessary for violence against women? There are a few interesting ideas here, but I would encourage the authors to create a more nuanced conceptual framework – where some factors may promote and others protect against perpetration of violence. Also some factors may produce violence acting alone (e.g. demands for dowry) and others in combination (social class plus attitudes towards women) to suggest increased likelihood of violence. The internal relationship between these identified factors needs to be reflected in the figure presented.

**Response: Thank you for the detailed comment. The revised conceptual framework has been provided which illustrates the risk factors and protective factors separately and the direct and combined effects on IPV.**

3. Strongly suggest that the figures and tables are labelled. It would also be good to have a table giving an overview of the relevant demographic information about the sample of participants in one glance.

**Response: The tables are labeled already as table 1, 2 and 3. The demographic information of the sample population is included in Table 1.**

4. Outcome variable for all types of violence focused only on violence by the husband, however, often violence (dowry related or otherwise) including sexual violence, is inflicted other members of the family – how do the authors account for that? At the very least some acknowledgement of the fact is required.

**Response: This is acknowledged in the limitations under discussion section.**

5. Similarly, they continue to say, “Similarly, violence had a significant positive association with the educational level of girls and wealth index.” – however it is a negative relationship between violence and educational level – i.e. the higher the education, the lower the violence and the same with wealth. The way ‘positive association’ reads now it implies, the greater the wealth the more the violence inflicted – which is the opposite of what the authors go on to explain is happening.

**Response: Authors apologize for their carelessness. The statement is modified highlighting a negative association in the revised manuscript.**

6. The authors report, “Interestingly, the likelihood of any violence was 41 percent and 2.06 times more likely among adolescent girls whose marital duration was 2-3 years [OR: 1.41; CI: 1.19-1.66] and 4 years or more [OR: 2.06; CI: 1.60- 2.64] respectively, compared to those whose marital duration was less than or equal to one year.” Why is this interesting? Is it not obvious – the more exposure, the more likelihood of experiencing violence? What would be interesting would be to explore if the trajectory of violence is upward with time and yet the girls choose to remain in the marriage? Is there a tipping point at which the girl might take some action or complain? Probably the data does not allow the authors to answer these questions, but they are nevertheless more interesting.

**Response: The word “Interestingly” is removed now. And yes, we agree with your comment that although the information is not available in UDAYA survey data, it would be interesting to explore if the trajectory of violence is upward with time and yet the girls choose to remain in the marriage.**

7. In the discussion section, the authors report: “In the process of empowering women, the previous study indicates that parents appear to be replacing property inheritance to their daughters by giving them alternative transfers in the form of higher dowries (42). This might have resulted in husbands and family in-laws of empowered women not perpetrating any violence.” – that is a big generalisation – often dowries are taken over by the in-laws and the woman has little or no control over her wealth. What is the evidence that higher dowries succeed in empowering women – especially if the amount bestowed is lower than that demanded by the in-laws?

**Response: Thank you for the comment. We agree that the statements can be misleading. The reported statements are removed now.**

8. Furthermore, the authors report, “The finding is similar to a study based on the National Family Health Survey which noted that because of being forthright against male dominance and better reporting of incidences of domestic violence, Indian working women have a higher likelihood of being victims of violence (47).”
It is not clear what the authors are claiming from their finding that those in paid employment are more likely to be victims of domestic violence – is it because they are more willing to acknowledge and report violence or because they pose a threat to their partners on traditional gender roles and hence are vulnerable to partner violence?

**Response: The statements are modified for better understanding as per suggestion. It reads now as “better reporting of incidences of domestic violence by which they pose a threat to their partners on traditional gender roles and hence, Indian working women are vulnerable to partner violence”.**

Further, does this mean that having paid employment makes some women more vulnerable and not having enough economic empowerment makes other women more vulnerable? If in both situations some women are vulnerable – then there must be other factors that will determine who is more likely to be vulnerable with economic empowerment and who isn’t. On the one hand the authors seem to suggest in the Discussion that when women are more likely to report violence (either because they are economically empowered or have been married for a longer period) they are more likely to suffer violence, but do not mention why this might be the case. Are the findings suggesting that women who are empowered to speak out against violence are more likely to suffer future violence as a result – if so – this sends out a very negative message as it does not support more empowerment for women – perhaps the authors might need to carefully caveat this finding.

**Response:** **We agree with the reviewer’s concerns. The following statements have been added to clarify this. “The current finding may be explained by the fact that economically empowered women who have more gender-conservative partners may have increased risk of experiencing violence especially, when they become less willing to conform to patriarchal norms in the household.” Relevant citation is provided.**

Further they go on to state that higher education status and social class act as protective factors and such women are less likely to be victims of violence. I am not sure the authors have reconciled these contrary findings effectively.

**Response: The discussion is revised accordingly. The added explanation reads as “The contrary findings in this study in terms of female labour-force participation increasing the risk and socioeconomic status reducing the risk of IPV highlight the importance of resource distribution and power imbalances within the family that may lead to or protect adolescent girls from IPV.” A related reference has been added too.**

9. A few grammatical issues need addressing. For example,
a. There are no page numbers for giving exact location, but in the results section the authors report “For instance, married girls faced more violence to increase the length of the marriage.” – which does not sound correct – Suggest rephrase.

**Response: The sentence is rephrased and corrected now.**

b. “A dearth of literature repeatedly found educational attainment and higher social status as major factors to reduce the likelihood of experiencing domestic violence in developed as well as developing countries (56–59)”. How can a dearth of literature (which means lack of literature) repeatedly find ….something?

**Response: The sentence modified by rephrasing the word “dearth” with “wealth”**

c. The authors state, “This study is based on sample of married girls from both the states which is 5,226 which is representative of 15-19 married girls at state level.” – perhaps they mean sample representing married girls between the age group of 15 to 19 years.

**Response: The statement is modified now for better understanding.**

10. One big gap in the paper is the relationship between violence and whether the woman has been able to produce children, preferably male offspring which might be related to or completely separate to demands for dowry. Were the women asked whether they felt their victimisation by their partner was due to unfulfilled demands for dowry and/or other factors?

**Response: This information was not collected in UDAYA.**

11. Did the dataset not have any information about dowry related domestic violence not perpetrated by the husband/partner? How do the authors propose to deal with this lacuna?

**Response: This information was not available.**

I do believe that the paper can make an important contribution if the authors work on developing a more nuanced conceptual framework and hypothesise which factors provide effective protection or which combination of factors make women more vulnerable to victimisation. A defined theoretical framework to make better sense of the data would make a significant contribution to the current literature on the topic. This would also help the authors structure their answer the ‘so what’ question better in terms of concrete policy implications which in the current version are a bit imprecise.

**Response: A more nuanced conceptual framework has been provided in the revised manuscript. The framework provides risk and protective factors of IPV. Discussion section has been revised substantially as per reviewer’s suggestions to explain the contrary findings and better understand the contexts.**
