# Supplementary material for: Dowry demand, perception of wife-beating, decision making power and associated partner violence among married adolescent girls: A cross-sectional analytical study in India
Source: PLoS One. 2024 Oct 24;19(10):e0312341. doi: 10.1371/journal.pone.0312341 (PMC11501023; doi:10.1371/journal.pone.0312341)
Supplement: Attachment — Submitted filename: Response to reviewers__Rev4__.docx [file pone.0312341.s006.docx]

**Response to reviewer comments**

**Reviewer 3**

I have no further comments except that the conceptual model needs to be updated and in line with the findings of the study. For example, non-Hindu category being only 15.9% of the sample and non-significant in Tables 2 and 3, is presented as a risk factor in the model- Domestic violence is prevalent across religions and is not a characteristic of one particular religion

**Response: Thank you for the additional comment and the recommendation. The conceptual framework has been modified as per suggestion.**

**Reviewer 5**

Thank you for the opportunity to review the manuscript entitled: “Dowry demand and associated partner violence among married adolescent girls: A cross-sectional analytical study in India”. The authors have tackled an important public health problem. Understanding how negative societal norms and cultures impact women’s risk of violence experience is important in the development of violence prevention programs and policies to mitigate these negative experiences, especially for young vulnerable women. However, I have some suggestions and recommendations that may help in strengthening the paper as outline below.

The authors have presented the conceptual framework for their study which seems rather simplistic. I expected more inter-linkages between the identifies clusters of risk factors or protective factors. As it is now, most factors only have direct paths to the outcome (IPV). What does literature say on link between Education status and attitudes that accept violence against women, decision making autonomy, employment etc for example. The other main concern is that their analytical approach does not seem to be consistent with their conceptual model.

**Response: Many thanks for the insightful comment. The conceptual framework is modified after incorporating the suggestions. The low level of education is kept as a risk factor of IPV and the higher level of education is included as a protective factor along with paid work for attitudes of accepting violence and decision making autonomy.**

Their “Data and Methods” section could be written in a more concise and clear way. The authors could briefly describe the original (UDAYA) study with references and then focus on describing data that they are using in this particular analysis. Otherwise, the whole first page of the “methods” section is confusing. For example, on the first page of the methods, they mention an effective sample size of 5226 and then on the second page the sample is 4893, with not so clear explanation on how the sample reduced to 4893. There is also a lot of repetitions of information in the section. The authors need to revise the section and make it more concise.

**Response: Thanks for the comment. Authors have made changes in the data and methods section and removed the repetitions of the information.**

It is not necessary to include the mathematical definition of a logistic model. If authors really want to include it, then they should interpret all the terms in the model in the context of their data. The authors have just given a generic interpretation of the linear component of the logit model but do not explain the logit function of the model in the context of their binary outcomes. It is easier to just state in the data analysis section that logistic regression model will be used. They do not need to include the model.

**Response: Thanks for the comment. Authors have removed mathematical definition of the logistic model.**

Authors should make it explicit in Table 3 that they are presenting adjusted OR. Considering that their write up in the results section for the logistic regression only covers combined violence outcome and not the specific type of violence, I would suggest Table 3 focuses on that. They could then include the crude ORs, so a reader could look at both crude and adjusted ORs for main exposure variables.

**Response: Dear reviewer, thank you for this comment. The authors have now mentioned that in the result section only combined violence was interpreted and not eh specific type of violence. Moreover, the ORs presented are adjusted odds ratios; however, the “adjusted” term was not earlier used. The authors have used the “aOR” for the adjusted odds ratio.**

The authors need to explain/justify in the methods section why the multivariable model included variables that were non-significant in the Chi-square test (Table 2).

**Response: Even though background characteristics(basic) were independently in-significant in the current study, the author still wants to consider age, relation & wealth index variables because adjusting these background characteristics may affect the significance of other determining/independent variables.**

Does the original UDAYA study have data on partner characteristics eg age of partner as these could also have impact on the power dynamics within the Household and impact on risk of IPV. If this data is available, authors need to adjust for these in the model. Another crucial variable that they could look at which would be more informative on cumulative risk of IPV (lifetime experience of IPV) than marriage duration is the age of participant at marriage. This could be derived from age of participant and marriage duration (assuming the participants are in their first marriage).

**Response: In the study, respondent age is from 15-19 Years which is already a very small span. Age at marriage is not normally distributed and there was high multi-collinearity (high VIF) between marriage duration and age at marriage. Hence, the authors did not consider including the age at marriage. The bivariate analysis showed no statistical significance in the association of partner age and IPV, thus not considered as well.**

The last sentence on second page of the discussion section: “The results are in agreement with an earlier study in India which found that women married for five years and longer were more likely to be beaten by their husbands [53].”, is rather misleading considering what is being measured in the study is ‘lifetime experience of IPV” as such the authors are looking at cumulative experience of IPV. I would suggest this sentence is revised to: “The results are in agreement with an earlier study in India which found that women married for five years and longer were more likely to have been beaten by their husbands [53].”, to reflect cumulative risk due cumulative exposure.

**Response: The comment is incorporated now. The statement is modified as per suggestion.**
