# Supplementary material for: Dowry demand, perception of wife-beating, decision making power and associated partner violence among married adolescent girls: A cross-sectional analytical study in India
Source: PLoS One. 2024 Oct 24;19(10):e0312341. doi: 10.1371/journal.pone.0312341 (PMC11501023; doi:10.1371/journal.pone.0312341)
Supplement: Attachment — Submitted filename: comments.docx [file pone.0312341.s007.docx]

Although suggested by reviewer 3 to present the odds ratios for crude ORs, the authors have not modified accordingly, additionally, I would recommend presenting the regression results by various forms of IPV and dowry demand (maybe in a separate table or in appendix)

In response to comments received from reviewer 3, the authors say that they included the background characteristics despite the variables being insignificant as they might have impact on IPV after being adjusted, however, in the next response the authors refute the idea of including age of partner claiming those variables to be insignificantly associated. I feel the authors should consider including partner’s age as it is an important determinant of IPV.

Abstract:

Line “The present study aimed to examine the association of dowry …” should be corrected to association between ‘. The conclusion section is unclear in terms of language and clarity.

The authors are advised to look thoroughly for grammatical errors.

Introduction- I do not understand what the authors mean by the statement “this study may contribute to how gender-specific risk and protective factors…” . I don’t feel that the analysis and context of the study in any-way related to both gender or gender-specific risk or protective factor.

Manuscript

In the introduction section the authors hypothesize that demand for dowry is associated with increased violence among married adolescent girls. However, the authors make no, make no mention of the rejection or acceptance of hypothesis. The authors should clearly mention it in the manuscript.

In data and methods, the authors mention that dowry demand question was asked as “Dowry demand by in-laws” however, in abstract and in results section the authors write as “dowry was demanded by their husbands”. The authors should check for such errors and correct it throughout the text.

The data section should be more concise.

As per the title of the manuscript, I expected a focus on the association and impact of dowry on the IPV which I could not find. An extensive elaborate discussion of other predictors impacting IPV diverts from the central idea of the paper. I would recommend the authors consider revising the discussion and an emphasis is given on the dowry which is the core idea of this paper. Additionally, the discussion should include findings from this study which now seems just to be a theoretical evidence from other literature.

Additionally, I could not find anything related to dowry and its association with dowry in the conclusion section which should have been as per the focus of the study.

I do not understand what the authors mean when they say” A large proportion reporting dowry may be explained by the fact that many of them do not consider dowry as a repressive practice but a rightful share…” If the respondent views the dowry as their rightful share, the proportion reporting dowry should be low. If so, what I understand and what is mentioned are two different sides of the coin.
