# Supplementary material for: Dowry demand, perception of wife-beating, decision making power and associated partner violence among married adolescent girls: A cross-sectional analytical study in India
Source: PLoS One. 2024 Oct 24;19(10):e0312341. doi: 10.1371/journal.pone.0312341 (PMC11501023; doi:10.1371/journal.pone.0312341)
Supplement: Attachment — Submitted filename: Plos one reviewer notes_5.30.23.docx [file pone.0312341.s008.docx]

**Review:**

Thank you very much for the opportunity to review this manuscript on IPV among married adolescent girls in India. This manuscript makes important contributions to understanding married adolescent girls’ risk of IPV related to dowry demand, acceptance of IPV, and household decision-making power. The large sample of married adolescents in India and the findings on the relationship between the factors (dowry demand, acceptance of IPV, and household decision-making power) and IPV are the strongest contributions of this paper to the literature on IPV risk. As such, major revisions focusing on these factors throughout the paper and analyses would greatly strengthen this manuscript and its value to the field.

**Abstract:**

Last sentence of “Background” missing word “using [a] large dataset”

Last sentence of “Conclusion” – suggest revising wording to clarify what “say” means. Is it decision-making power or agency?

**Background:**

The background provided on dowry demand and hypothesized relationship with IPV is strong and some of the context of gender inequity is provided that helps frame why they authors included the other exposure variables. However, the background on the other exposure variables and the focus on these in the analysis needs to be reflected in this background more. As commented below about Figure 1, the background section could be strengthened with a more structured approach to discussing the main exposure variables that will be focused on in this paper and are contributions to the literature: dowry demand (justification is already quite clear), perception of wife-beating, decision-making on work and household purchases.

The first paragraph talks about IPV and dowry broadly/globally, but then provides very specific information about legal codes in India. The setting of your paper needs to be introduced more formally – whether you want to frame it around South Asia or India is your choice. I would recommend introducing the setting in this first paragraph – talk about IPV globally and then particularly in India. Next paragraph, talk about dowry broadly and then in India specifically.

Is there a national estimate for how common dowries are? Please provide, if so, as this would provide a parallel description with the IPV estimates.

Last sentence in paragraph 3: I recommend rewording for clarity, “…could be a major determinant for domestic violence victimization.”

Fifth paragraph: Please define dowry deaths. Also, please reword this sentence for clarity, “However, little is known about the nature of the problem and married adolescent girls' experiences of dowry demand during and early years of their marriage resulting in some form of violence”. Particularly what is meant by “during and early years of their marriage” and what is the following clause attached to in this sentence: “some form of violence.”

Please correct small grammatical errors throughout.

Figure 1: Overall, this conceptual framework does not add a lot of value to this manuscript and may cause more confusion than benefit. Because the analysis does not align with this framework, the authors may benefit more from very clearly justifying in the background the inclusion of the key hypothesized exposures for IPV: dowry demand (justification is already quite clear), perception of wife-beating, decision-making on work and household purchases.

If the authors do decide to keep it, here are some specific concerns: 1) the analysis does not reflect the relationship presented here. 2) Paid work and higher education should appear further to the left as it proceeds justification for wife beating and decision-making autonomy. 3) What is meant by box that says, “Can be catalized by…”? Meaning dowry demand by in-laws plus being of lower wealth, lower caste, and rural may make IPV more likely? If you’re describing a causal ordered process, seems like there should be an arrow lining dowry demand to your other box on rural, caste, and income, and then continue to IPV. Alternatively, is caste/income/rural a driver of dowry demand? If so, that box should come before the dowry demand one on that line. 4) Same comment for “Justified perception over wife beating” (this should also be corrected to read: “Perceived justification for wife beating”).

**Methods**

Data

Paragraph 1 needs further refinement for clarity. Seems to me that the necessary information is here the order of it just needs to be adjusted. My recommendation would be to revise the first paragraph after the third sentence so that information is provided in the following order (mainly rearrange sentences and take out anything extra):

1. Sampling frame
2. Total sample of PSUs
3. Factors included in stratified sampling and their hierarchy
4. What was done for rural villages
5. What was done for urban villages

Then I would recommend making the information about the five categories of respondents a separate paragraph.

Measures

Outcome variable - First two sentences are repetitive. Delete first sentence and edit the second sentence to read, “Emotional violence was measured using the question “Did your husband ever do something to humiliate you in front of others or threatened you to hurt or harm someone close to you?” and the response was coded as no (0) and yes (1).

What were the original response options on the violence outcome questions before recoding?

Exposure variable – This paragraph could use some tightening (make more succinct) I would recommend starting the paragraph with the question(s) that were asked about dowry demand. Were these two separate questions or combined as written in the text? Also, what were the response options? Then state how the variable was created.

I would recommend that sentences 3-5 of the “exposure variables” paragraph be removed, as these should be already established in your introduction. I would recommend removing and go right into defining your other exposure variables. The definition of each variable would be clarified if a paragraph for each variable was included. That paragraph could briefly include the question that was asked, where did that measure come from with citation (previously validated? Used elsewhere? New and if so, how it was developed?), the response options, and how the variable was created/coded.

For the variable about decisions regarding household purchases, it is important that throughout the paper this be referred to as household “purchases” (not “purchase”) because the current phrasing throughout the paper makes this sounds like it is about the decision to buy a house or not, rather than everyday household purchases. This edit is needed throughout the paper and tables.

Statistical analysis

Is there a citation for the UDAYA dataset documentation/information about weights?

I recommend that the statement about terming late adolescents to be removed because this term is not used at all in the majority of the paper.

What were the adjusted models controlling for? Or were they only adjusted for clustering and stratum effects? Rather than considering marital duration as an exposure variable, I would be more inclined to control for this variable. It seems logical and well-established that longer duration of marriage would provide more opportunity and increased risk for violence within marriage so feels less like a novel finding and more like an important variable to control for. Similarly, age (exclude if collinear with marriage duration), urban/rural residence, religion, caste, education, wealth, and state are all demographic characteristics with known patterns of risk for IPV that are better suited as covariates. If the authors were to control for these variables, it would help to isolate the estimated effects of dowry demand, perception of wife-beating, decision-making on work and household purchases on IPV risk, which is the strongest contribution of this paper.

Did you consider controlling for age at marriage? There is evidence to suggest that risk for violence varies (as well as many of the exposure variables) by how young the adolescent is when she marries. Along these lines, did you consider (or was it possible) to control for the age difference between the wife and husband? Again, evidence suggests that this might be an important indicator of the power imbalance between husband and wife, and therefore be related to increased risk for violence.

**Results**

The contribution of this paper are the results related to the estimated effect of dowry demand, perception of wife-beating, decision-making on work and household purchases on IPV risk. As such, I recommend keeping Tables 1 and 2 as they are (as well as the presentation of results), and modifying Table 3 to include analyses that are focused on these 4 exposures with aOR results that are adjusted for the sociodemographic factors.

More detailed comments on current manuscript results section:

The sentence is a bit misleading or at the very least, confusing: “Moreover there was a positive relationship between type of violence and duration…” Put more simply, the authors could say what types of violence are associated with marital duration and exclude the example.

Paragraph 3, sentence 5: Recommend revising for clarity. In particular, I would recommend rephrasing to “adolescent girls whose decisions about their work are made by others” (similar edit to other instances in this paragraph referring to “decisions taken”) and removing the work “only” to remove the interpretation in this statement.

Par 3, sentence 7: It is a bit odd to interpret the 1.41 aOR as a percent and the 2.06 as a likelihood in the same sentence. I would recommend choosing one approach for both within a sentence.

Results related to caste: clarify that “other castes” does not include OBC.

Table 3: footnotes should clarify what the aOR models adjusted for.

**Discussion**

Paragraph 2: It seems likely that women who have experienced IPV are more likely to perceive violence is justified because of the ways that IPV perpetrators rationalize and tell their victims that they are deserving of that violence, rather than their pre-existing acceptance of inequitable gender roles being the cause of their husbands’ violence. Many survivors of IPV are told so many times by their perpetrators that they are deserving of the abuse that the survivor begins to believe it themselves, or at least accepts this perception to cope with the need to remain married to the person. The discussion of this factor might benefit from a more nuanced discussion of the direction of this relationship being interrogated and bring in literature to help frame this discussion. This discussion could replace some of the discussion about the sociodemographic variables being associated with IPV that are less of a strong contribution to the literature.

Limitations – the discussion of recall and social desirability bias would benefit from an indication of which direction the authors hypothesize the bias to appear and a justification for why they think this bias might occur in that direction.

The last limitation mentioned is not clear – please further clarify what additional types of studies are needed. Are the authors referring to mechanistic studies about how early marriage leads to IPV? Qualitative work?

Conclusion: While an important set of recommendations, I would recommend for the authors to keep their recommendations more connected to the results identified in this study and not go further. For example, results do not offer insight on how reproductive health services should be conducted so I would recommend removing. Additionally, recommendations about women’s employment seem to overlook the previous discussion about how female employment can also be a risk factor for violence.

Overall, the conclusion could really benefit from focusing on the context of gender inequity and how gender inequity, as expressed through dowry demand, acceptance of IPV, and lack of female decision-making power, could be better addressed within society.
