# Supplementary material for: Dowry demand, perception of wife-beating, decision making power and associated partner violence among married adolescent girls: A cross-sectional analytical study in India
Source: PLoS One. 2024 Oct 24;19(10):e0312341. doi: 10.1371/journal.pone.0312341 (PMC11501023; doi:10.1371/journal.pone.0312341)
Supplement: Attachment — Submitted filename: Plos one reviewer memo_Rev5_.docx [file pone.0312341.s009.docx]

**Response to comments**

**Reviewer #6**

Thank you very much for the opportunity to review this manuscript on IPV among married adolescent girls in India. This manuscript makes important contributions to understanding married adolescent girls’ risk of IPV related to dowry demand, acceptance of IPV, and household decision-making power. The large sample of married adolescents in India and the findings on the relationship between the factors (dowry demand, acceptance of IPV, and household decision-making power) and IPV are the strongest contributions of this paper to the literature on IPV risk. As such, major revisions focusing on these factors throughout the paper and analyses would greatly strengthen this manuscript and its value to the field.

**Abstract:**

Last sentence of “Background” missing word “using [a] large dataset”.

**Response**: *Dear reviewer, thank you for your feedback. We have added the missing word "using a large dataset" in the revised manuscript.*

Last sentence of “Conclusion” – suggest revising wording to clarify what “say” means. Is it decision-making power or agency?

**Response**: *Dear reviewer, thank you for your comment. The authors have addressed the concern by revising the conclusions part of the abstract.*

**Background:**

The background provided on dowry demand and hypothesized relationship with IPV is strong and some of the context of gender inequity is provided that helps frame why they authors included the other exposure variables. However, the background on the other exposure variables and the focus on these in the analysis needs to be reflected in this background more. As commented below about Figure 1, the background section could be strengthened with a more structured approach to discussing the main exposure variables that will be focused on in this paper and are contributions to the literature: dowry demand (justification is already quite clear), perception of wife-beating, decision-making on work and household purchases.

The first paragraph talks about IPV and dowry broadly/globally, but then provides very specific information about legal codes in India. The setting of your paper needs to be introduced more formally – whether you want to frame it around South Asia or India is your choice. I would recommend introducing the setting in this first paragraph – talk about IPV globally and then particularly in India. Next paragraph, talk about dowry broadly and then in India specifically.

Is there a national estimate for how common dowries are? Please provide, if so, as this would provide a parallel description with the IPV estimates.

Last sentence in paragraph 3: I recommend rewording for clarity, “…could be a major determinant for domestic violence victimization.”

Fifth paragraph: Please define dowry deaths. Also, please reword this sentence for clarity, “However, little is known about the nature of the problem and married adolescent girls' experiences of dowry demand during and early years of their marriage resulting in some form of violence”. Particularly what is meant by “during and early years of their marriage” and what is the following clause attached to in this sentence: “some form of violence.”

Please correct small grammatical errors throughout.

**Response**: *We apologize for these errors and have corrected them in the updated version of the manuscript. We now provide a national estimate of dowry demand in India in the revised introduction part. We have implemented your other suggestions, reorganizing the manuscript to give prominence to global IPV studies, followed by a focus on South Asia. The paper now centers on two key contributions: examining perceptions of wife-beating and exploring decision-making dynamics related to work and household purchases. The introduction introduces the setting more formally, first discussing IPV globally and then specifically in India. The discussion on dowry follows a recommended structure in the updated version of the manuscript.*

Figure 1: Overall, this conceptual framework does not add a lot of value to this manuscript and may cause more confusion than benefit. Because the analysis does not align with this framework, the authors may benefit more from very clearly justifying in the background the inclusion of the key hypothesized exposures for IPV: dowry demand (justification is already quite clear), perception of wife-beating, decision-making on work and household purchases.

If the authors do decide to keep it, here are some specific concerns: 1) the analysis does not reflect the relationship presented here. 2) Paid work and higher education should appear further to the left as it proceeds justification for wife beating and decision-making autonomy. 3) What is meant by box that says, “Can be catalized by…”? Meaning dowry demand by in-laws plus being of lower wealth, lower caste, and rural may make IPV more likely? If you’re describing a causal ordered process, seems like there should be an arrow lining dowry demand to your other box on rural, caste, and income, and then continue to IPV. Alternatively, is caste/income/rural a driver of dowry demand? If so, that box should come before the dowry demand one on that line. 4) Same comment for “Justified perception over wife beating” (this should also be corrected to read: “Perceived justification for wife beating”).

**Response:** *We appreciate this suggestion and have now removed the conceptual framework to avoid confusion among readers!*

**Methods**

**Data**

Paragraph 1 needs further refinement for clarity. Seems to me that the necessary information is here the order of it just needs to be adjusted. My recommendation would be to revise the first paragraph after the third sentence so that information is provided in the following order (mainly rearrange sentences and take out anything extra):

1. Sampling frame
2. Total sample of PSUs
3. Factors included in stratified sampling and their hierarchy
4. What was done for rural villages
5. What was done for urban villages

Then I would recommend making the information about the five categories of respondents a separate paragraph.

**Response**: *Thank you for the comment! Per comments from Reviewer #7, we have reduced the content of the data section to make it concise and provided a citation for further information on survey sampling procedures.*

**Measures**

Outcome variable - First two sentences are repetitive. Delete first sentence and edit the second sentence to read, “Emotional violence was measured using the question “Did your husband ever do something to humiliate you in front of others or threatened you to hurt or harm someone close to you?” and the response was coded as no (0) and yes (1). What were the original response options on the violence outcome questions before recoding?

**Response**: *Thank you for your thoughtful suggestions. And the violence variable consists of 7 questions. If the respondent ever expressed any of the following and responded "sometimes" or "often" in the last 12 months, we recorded ever experiencing those into 1 as 'yes' and never experiencing them into 0 as ‘no.’*

*During the survey, the following questions were asked:*

1. *Did your husband ever do any of the following to you: YES/NO*
2. *How often did this happen in the last 12 months: never, sometimes, or often? a) slap you? b) twist your arm or pull your hair? c) push you, shake you, or throw something at you? d) punch you with his fist or with something that could hurt you? e) kick you, drag you, or beat you up? f) try to choke you or burn you on purpose? g) threaten or attack you with a knife, gun, or any other weapon?: NEVER/SOMETIMES/OFTEN*

Exposure variable – This paragraph could use some tightening (make more succinct) I would recommend starting the paragraph with the question(s) that were asked about dowry demand. Were these two separate questions or combined as written in the text? Also, what were the response options? Then state how the variable was created.

**Response**: *Dear Reviewer, the author has implemented your suggestions. Dowry demand by in-laws was assessed by asking, “Has anybody in your husband’s family ever said that the dowry/gift/cash you brought was too little or that you did not bring anything?” the response was recoded as 1 ('yes') and 0 ('no') otherwise.*

*The question was singular, with only one inquiry and response options limited to 'Yes' and 'No,' as demonstrated above. This is clarified now in the updated version.*

I would recommend that sentences 3-5 of the “exposure variables” paragraph be removed, as these should be already established in your introduction. I would recommend removing and go right into defining your other exposure variables. The definition of each variable would be clarified if a paragraph for each variable was included. That paragraph could briefly include the question that was asked, where did that measure come from with citation (previously validated? Used elsewhere? New and if so, how it was developed?), the response options, and how the variable was created/coded.

**Response**: *Dear Reviewer, the author has implemented your suggestions. We appreciate your suggestions.*

For the variable about decisions regarding household purchases, it is important that throughout the paper this be referred to as household “purchases” (not “purchase”) because the current phrasing throughout the paper makes this sounds like it is about the decision to buy a house or not, rather than everyday household purchases. This edit is needed throughout the paper and tables.

**Response**: *Thank you for pointing out. Made the changes.*

*The inquiry pertained to household purchases: "Who primarily decides about major household purchases? Do you alone decide, jointly decide with others, or do others alone decide?" In the Results and other sections, it was corrected from "purchase" to accurately reflect the question*.

**Statistical analysis**

Is there a citation for the UDAYA dataset documentation/information about weights?

**Response**: *Yes, the relevant citation for the UDAYA dataset documentation and the survey weights is provided now in the data section.*

I recommend that the statement about terming late adolescents to be removed because this term is not used at all in the majority of the paper.

**Response**: *Dear reviewer, thank you for the comment. The authors have addressed the concern by removing the term "late adolescents" from the manuscript.*

What were the adjusted models controlling for? Or were they only adjusted for clustering and stratum effects? Rather than considering marital duration as an exposure variable, I would be more inclined to control for this variable. It seems logical and well-established that longer duration of marriage would provide more opportunity and increased risk for violence within marriage so feels less like a novel finding and more like an important variable to control for. Similarly, age (exclude if collinear with marriage duration), urban/rural residence, religion, caste, education, wealth, and state are all demographic characteristics with known patterns of risk for IPV that are better suited as covariates. If the authors were to control for these variables, it would help to isolate the estimated effects of dowry demand, perception of wife-beating, decision-making on work and household purchases on IPV risk, which is the strongest contribution of this paper.

**Response**: *Thank you for your suggestions. To clarify, our models were adjusted for these demographic characteristics to isolate the estimated effects of dowry demand, perception of wife-beating, and decision-making on work and household purchases on IPV risk. These are clarified as table notes in the revised version. The tables are modified per reviewer suggestions in the updated version.*

Did you consider controlling for age at marriage? There is evidence to suggest that risk for violence varies (as well as many of the exposure variables) by how young the adolescent is when she marries. Along these lines, did you consider (or was it possible) to control for the age difference between the wife and husband? Again, evidence suggests that this might be an important indicator of the power imbalance between husband and wife, and therefore be related to increased risk for violence.

**Response**: *Dear reviewer, in light of your suggestion and considering the recommendations of other reviewers, we revisited the analysis and included controls for the age of the spouse. Thank you for bringing this to our attention, and we value your input in enhancing the robustness of our study.*

**Results**

The contribution of this paper are the results related to the estimated effect of dowry demand, perception of wife-beating, decision-making on work and household purchases on IPV risk. As such, I recommend keeping Tables 1 and 2 as they are (as well as the presentation of results), and modifying Table 3 to include analyses that are focused on these 4 exposures with aOR results that are adjusted for the sociodemographic factors.

**Response:** *Dear reviewer, thanks for the comment. The authors have amended the table-3 (and table 4 in the updated version) and the results as per your valuable suggestion.*

More detailed comments on current manuscript results section:

The sentence is a bit misleading or at the very least, confusing: “Moreover there was a positive relationship between type of violence and duration…” Put more simply, the authors could say what types of violence are associated with marital duration and exclude the example.

**Response**: *As per your previous comment we have shortened the results for table 3 (and table 4 in the updated version), therefore, this sentence is not present in the manuscript now. However, thank you so much for your in-depth suggestion.*

Paragraph 3, sentence 5: Recommend revising for clarity. In particular, I would recommend rephrasing to “adolescent girls whose decisions about their work are made by others” (similar edit to other instances in this paragraph referring to “decisions taken”) and removing the work “only” to remove the interpretation in this statement.

**Response**: *Dear reviewer, thanks for the comment. The authors have amended the sentences as per your comment***.**

Par 3, sentence 7: It is a bit odd to interpret the 1.41 aOR as a percent and the 2.06 as a likelihood in the same sentence. I would recommend choosing one approach for both within a sentence.

**Response**: *As per your previous comment we have shortened the results for table 3 (and table 4 in the updated version), therefore, this sentence is not present in the manuscript now. However, thank you so much for your in-depth suggestion, we have cautioned this while interpreting the results in the updated manuscript***.**

Results related to caste: clarify that “other castes” does not include OBC.

**Response**: *As per your previous comment we have shortened the results for table 3, therefore, this sentence is not present in the manuscript now.*

Table 3: footnotes should clarify what the aOR models adjusted for.

**Response**: *Dear reviewer, thanks for the comment. The authors have amended the footnote of the table 3 (and table 4 in the updated version) as per your suggestion.*

**Discussion**

Paragraph 2: It seems likely that women who have experienced IPV are more likely to perceive violence is justified because of the ways that IPV perpetrators rationalize and tell their victims that they are deserving of that violence, rather than their pre-existing acceptance of inequitable gender roles being the cause of their husbands’ violence. Many survivors of IPV are told so many times by their perpetrators that they are deserving of the abuse that the survivor begins to believe it themselves, or at least accepts this perception to cope with the need to remain married to the person. The discussion of this factor might benefit from a more nuanced discussion of the direction of this relationship being interrogated and bring in literature to help frame this discussion. This discussion could replace some of the discussion about the sociodemographic variables being associated with IPV that are less of a strong contribution to the literature.

**Response**: *After the second paragraph in the discussion section, we have touched upon the strategies employed by IPV perpetrators to convince survivors that they are deserving of the abuse they endure. We further emphasize the profound influence these manipulative tactics can have on a survivor's self-perception and psychological well-being.*

Limitations – the discussion of recall and social desirability bias would benefit from an indication of which direction the authors hypothesize the bias to appear and a justification for why they think this bias might occur in that direction.

**Response**: *We have discussed how recall bias and social desirability bias may lead to an underestimation of the violence experienced by married adolescent girls. Furthermore, we provided a justification for why these biases may manifest specifically in this manner.*

The last limitation mentioned is not clear – please further clarify what additional types of studies are needed. Are the authors referring to mechanistic studies about how early marriage leads to IPV? Qualitative work?

**Response**: *We have clarified the type of future studies needed i.e those employing mechanistic qualitative studies, such as in-depth interviews, focus groups, and participant observation, to explore the link between early marriage amongst adolescent girls and IPV.*

Conclusion: While an important set of recommendations, I would recommend for the authors to keep their recommendations more connected to the results identified in this study and not go further. For example, results do not offer insight on how reproductive health services should be conducted so I would recommend removing. Additionally, recommendations about women’s employment seem to overlook the previous discussion about how female employment can also be a risk factor for violence.

**Response**: *We have made the necessary changes by revising the recommendations to ensure they are more closely aligned with the study result*

Overall, the conclusion could really benefit from focusing on the context of gender inequity and how gender inequity, as expressed through dowry demand, acceptance of IPV, and lack of female decision-making power, could be better addressed within society.

**Response**: *In response to the reviewer's suggestion, we have refined the conclusion to center more explicitly on the theme of gender inequity*

*Thank you for taking your time for reviewing our manuscript, we are grateful for your time!*

Reviewer #7

Responses to 2, 3, 5 reflect that overall, the manuscript is well written and analyses are strong, but improvements are needed on all of these areas.

Although suggested by reviewer 3 to present the odds ratios for crude ORs, the authors have not modified accordingly, additionally, I would recommend presenting the regression results by various forms of IPV and dowry demand (maybe in a separate table or in appendix)

**Response**: *Dear Reviewer, we apologize for overlooking some of the previous comments. We have now implemented your suggestions and added the Crude ORs table in the updated version and detailed tables in the Supplementary file.*

In response to comments received from reviewer 3, the authors say that they included the background characteristics despite the variables being insignificant as they might have impact on IPV after being adjusted, however, in the next response the authors refute the idea of including age of partner claiming those variables to be insignificantly associated. I feel the authors should consider including partner’s age as it is an important determinant of IPV.

**Response**: *Dear reviewer, considering your suggestion and the recommendations of other reviewers, we revisited the analysis and included controls for the age of the spouse. Thank you for bringing this to our attention, and we value your input in enhancing the robustness of our study.*

Abstract:

Line “The present study aimed to examine the association of dowry …” should be corrected to association between ‘. The conclusion section is unclear in terms of language and clarity.

The authors are advised to look thoroughly for grammatical errors.

Introduction- I do not understand what the authors mean by the statement “this study may contribute to how gender-specific risk and protective factors…” . I don’t feel that the analysis and context of the study in any-way related to both gender or gender-specific risk or protective factor.

**Response**: *Thank you for your feedback. We have corrected the line in the abstract. Additionally, we appreciate your observation about the conclusion section, and work on improving its language and clarity during the revision process.*

*Regarding the Introduction, we understand your concern about the statement "this study may contribute to how gender-specific risk and protective factors…". We agree that the analysis and context of the study may not appear directly related to gender or gender-specific factors. We have revised the statement to better align with the study's focus and provide a clearer explanation in the revised manuscript. Thank you for bringing these points to our attention, and we have made the necessary adjustments to enhance the overall quality of the study.*

Manuscript

In the introduction section the authors hypothesize that demand for dowry is associated with increased violence among married adolescent girls. However, the authors make no, make no mention of the rejection or acceptance of hypothesis. The authors should clearly mention it in the manuscript.

**Response**: *Dear Reviver, thank you for highlighting the lack of clarity on the hypothesis outcome in the introduction. In the revised manuscript, we addressed this by modifying the hypothesis to reflect our objectives and by stating that the findings supported our hypothesis in the discussion.*

In data and methods, the authors mention that dowry demand question was asked as “Dowry demand by in-laws” however, in abstract and in results section the authors write as “dowry was demanded by their husbands”. The authors should check for such errors and correct it throughout the text.

**Response:** *Dear reviewer, we apologize for this oversight on our part and thank you for the valuable comment. We have made the changes throughout the manuscript, wherever needed.*

The data section should be more concise.

**Response**: *Absolutely, done!*

As per the title of the manuscript, I expected a focus on the association and impact of dowry on the IPV which I could not find. An extensive elaborate discussion of other predictors impacting IPV diverts from the central idea of the paper. I would recommend the authors consider revising the discussion and an emphasis is given on the dowry which is the core idea of this paper. Additionally, the discussion should include findings from this study which now seems just to be theoretical evidence from other literature.

**Response**: *We appreciate your suggestions, thank you! We have revised the discussion section per your suggestions and focused on our key findings.*

Additionally, I could not find anything related to dowry and its association with dowry in the conclusion section which should have been as per the focus of the study.

**Response**: *This has been addressed as we have better explained the findings of this study within the context of how dowry practices may exacerbate gender inequality through the manifestations of dowry demands, acceptance of intimate partner violence (IPV), and the absence of decision-making power among women.*

I do not understand what the authors mean when they say” A large proportion reporting dowry may be explained by the fact that many of them do not consider dowry as a repressive practice but a rightful share…” If the respondent views the dowry as their rightful share, the proportion reporting dowry should be low. If so, what I understand and what is mentioned are two different sides of the coin.

**Response**: *We have excluded this sentence from the discussion to offer more clarity*.

*Thank you for taking your time for reviewing our manuscript, we are grateful!*
