# Supplementary material for: Dowry demand, perception of wife-beating, decision making power and associated partner violence among married adolescent girls: A cross-sectional analytical study in India
Source: PLoS One. 2024 Oct 24;19(10):e0312341. doi: 10.1371/journal.pone.0312341 (PMC11501023; doi:10.1371/journal.pone.0312341)
Supplement: Attachment — Submitted filename: Reviewer Memo_Rev6_.docx [file pone.0312341.s010.docx]

**Response to comments**

**Reviewer #8**

Background

Under the first sentence in the 2nd paragraph under "Background", the sentence "In South Asia, IPV is a complex challenge that is related to cultural dynamics." could be strengthened further by stating some of these cultural dynamics and possibly stating references that validates these cultural determinants with IPV.

*Response: We appreciate your comment! We have added more details and validating references per suggestion.*

In the subsequent sentence in the same paragraph, it would be great to start the sentence with an opening that establishes the role of personal experiences and societal factors in amplifying the likelihood of IPV. This can come just before the sentence "Research links IPV in the region, including India....".

*Response: We have updated the text and elaborated on the role of personal experiences and societal factors in amplifying the likelihood of IPV.*

Furthermore, within the same paragraph (sentence before last), the use of the word "ecology of IPV" seems vague - consider alternative terms like "prevalence".

*Response: We have replaced the word with “prevalence”.*

Last sentence in the second paragraph, "Additionally, interventions, such as couple-based programs, have been explored for the primary prevention of IPV in India, emphasizing the need for effective strategies to address this issue" provided valuable perspective. However, the author should either expand on these interventions briefly here or move this entire sentence to the Conclusion session and share some existing interventions as suggested here.

*Response: We have expanded on these interventions in the updated manuscript.*

Data & Method

I would re-label this section to "Methodology" and then the "data' to 'Overall Approach' - First paragraph and then "Sampling Strategy" - starting with the 2nd paragraph. Under the first paragraph, consider including a sentence briefly describing what the UDAYA project is about.

*Response: Thank you for your comment! We have updated the section heading and added a brief description of UDAYA project under the first paragraph on the Overall Approach.*

Variable description -

Include a summary paragraph that briefly described the types of variables analysed (in this case outcome & exposure variable) and a short rationale for focusing on each.

*Response: We have added a description of types of variables analyzed and a short rationale for each.*

Statistical Analysis -

In line 3, you established that association between the binary 'outcome variable' and 'explanatory variables' were conducted. However, in your description of variables, you used 'exploratory variable' - there is need for consistency.

*Response: We have updated the text and used the word “explanatory” throughout the manuscript.*

Discussion -

Last sentence in paragraph 5 should be reworded for clarity from "The present study in concordance with this found that higher education and upper social class in the Indian caste hierarchy of married adolescent girls were protective factors against partner violence" to "The findings from this study which establishes that higher education and upper social class in the Indian caste hierarchy of married adolescent girls were protective factors against partner violence, is consistent with existing literature.

*Response: Incorporated this suggestion in the updated version. Thank you for pointing this out!*

The subsequent paragraph (2nd sentence seem incomplete: "Owing to the cross-sectional nature of this study, no causal relationships could be established"...add "between X variables and Y variables". You may decide to stratify which of the causal factors were worse off between the personal, social, cultural or otherwise.

*Response: Thank you for your valuable feedback. We have revised the manuscript to specify the variables involved in the analysis. Instead of the general statement that "no causal relationships could be established," we now clarify that "no causal relationships between dowry demand, perception of wife-beating, decision making power and IPV could be established." Regarding the suggestion to stratify the causal factors, while this study did not explicitly examine the stratification of personal, social, and cultural factors influencing intimate partner violence (IPV), we have included a discussion of key studies in the literature that explore the comparative impact of these factors. This provides context and supports the interpretation of our findings within the broader framework of existing research.*
